# Alcohol Consumption and the Risk of Cancer: A Meta-Analysis

**Published:** 2001

**Authors:** Vincenzo Bagnardi, Marta Blangiardo, Carlo La Vecchia, Giovanni Corrao

**Affiliations:** Vincenzo Bagnardi, Ms.C., and Marta Blangiardo, Ms.C., are Ph.D. students in statistics, and Giovanni Corrao, Ph.D., is a full professor in statistics at the Department of Statistics, University of Milan-Bicocca, Milan, Italy. Carlo La Vecchia, M.D., is an associate professor in epidemiology at the Institute of Medical Statistics and Biometry, University of Milan, and head of the laboratory of general epidemiology at the Mario Negri Institute of Pharmacological Research, Milan, Italy

**Keywords:** AOD (alcohol or other drug) consumption, chronic AODE (effects of AOD use, abuse, and dependence), cancer, carcinogenesis, oral disorder, esophageal disorder, dose-response relationship, gender differences, tobacco in any form, distilled alcoholic beverage, drug concentration, risk analysis, meta-analysis

## Abstract

Alcohol consumption has been linked to an increased risk for various types of cancer. A combined analysis of more than 200 studies assessing the link between alcohol and various types of cancer (i.e., a meta-analysis) sought to investigate this association in more detail. This meta-analysis found that alcohol most strongly increased the risks for cancers of the oral cavity, pharynx, esophagus, and larynx. Statistically significant increases in risk also existed for cancers of the stomach, colon, rectum, liver, female breast, and ovaries. Several mechanisms have been postulated through which alcohol may contribute to an increased risk of cancer. Concurrent tobacco use, which is common among drinkers, enhances alcohol’s effects on the risk for cancers of the upper digestive and respiratory tract. The analysis did not identify a threshold level of alcohol consumption below which no increased risk for cancer was evident.

Regular alcohol consumption can have numerous consequences, beneficial or detrimental, on the health of the drinker. For example, light-to-moderate alcohol consumption^1^ may protect against certain types of heart disease and stroke. Conversely, heavy drinking has been associated with liver disease; cardiovascular disease; disorders of the digestive tract; and illness or death from alcohol-related injuries, motor vehicle crashes, and violence. Another group of disorders that has been linked to drinking is cancer, particularly cancers of the upper airway and digestive tract (e.g., mouth, pharynx, larynx, and esophagus). Although alcohol has not been shown to cause cancer (i.e., be carcinogenic) in animal studies, strong epidemiological evidence indicates that consumption of alcoholic beverages increases the risk of those cancers. Alcohol consumption also is associated with primary liver cancer. This relationship is difficult to investigate in epidemiological studies, however, because it is more indirect. Thus, alcohol causes cirrhosis of the liver in a substantial proportion of heavy drinkers, which then can lead to liver cancer. In addition, heavy alcohol consumption can increase the drinker’s risk for infection with the hepatitis C virus (HCV), which in turn can also result in liver cancer (for more information on the relationship between alcohol consumption and HCV infection, see the article in this issue by Lieber, pp. 245–254).

Alcohol consumption also has been linked to cancers of the large bowel (i.e., colon and rectum) in both men and women and to breast cancer in women, although these associations have not yet been proven unequivocally. Nevertheless, because these are the two most common types of cancer in developed countries after lung cancer, even a moderate increase in risk may result in a relatively large number of additional cases and therefore have important public health implications. The association between alcohol consumption and other types of cancer (e.g., stomach, pancreatic, prostate, and endometrial cancer) is still controversial (International Agency for Research on Cancer [IARC] 1988; Doll et al. 1999).

The increased risk of cancer among heavy drinkers is primarily attributed to the alcohol (chemically referred to as ethanol) in alcoholic beverages. Thus, the risk tends to increase with the overall amount of ethanol consumed. It is still unclear, however, whether any defined consumption threshold exists below which no increased risk for cancer is evident (IARC 1988; Doll et al. 1999).

To evaluate the overall effects of alcohol on the cancer risk of a population, one must accurately quantify its effects on various types of tumors. To this end, researchers have performed comprehensive meta-analyses of published studies investigating the relationship between alcohol intake and the risk for numerous types of cancer. Meta-analyses are studies that pool data from several studies, thereby substantially enhancing the overall number of cases evaluated. This approach allows researchers to detect relationships that may have been overlooked in the individual studies because of the relatively small sample size and insufficient statistical power of those individual studies. This article summarizes the major findings of one such meta-analysis (Corrao et al. 1999, 2000).

## Methods Used for the Meta-Analysis

To identify scientific articles to include in the meta-analysis, the researchers followed three steps: First, they conducted a search of several bibliographic databases (e.g., *MEDLINE*, *Current Contents*, *EMBASE*, *CAB Abstracts,* and *Core Biomedical Collection*) for studies published between 1966 and 2000. Second, the investigators reviewed all references in the resulting articles to identify any studies that had not been found in the database search. And third, to ensure that the list of studies included was as complete as possible, the investigators conducted a manual search of the most relevant journals of epidemiology and medicine and compared their search results with those of other general reviews and meta-analyses published on this topic.

Each article identified by this search process was reviewed and included in the analysis if it met the following three criteria:

- The article was a case-control or a cohort study published as an original article. Case-control studies compare people with a certain disease (e.g., cancer)—the cases—with a similar group of people without the disease— the controls. Investigators then collect information (e.g., regarding alcohol consumption) for both groups to determine whether differences exist between the groups. For example, significant differences between the groups with respect to alcohol intake suggest that alcohol was a causative factor in the disease. Cohort studies follow a group of initially healthy people over a prolonged period of time. At the outset, the investigators determine the participants’ drinking patterns so that they can relate drinking patterns and disease development during the followup period.
- The findings of the study were expressed as relative risk (RR) or odds ratio (OR) and considered at least three levels of alcohol consumption. The RR determines the strength of the relationship between a variable (e.g., alcohol consumption) and the development of a disease. The RR for a disease among people without the variable (e.g., among abstainers) is defined as 1.0. A RR among people with the variable (e.g., among drinkers) of greater than 1.0 indicates that the variable increases the risk of the disease. Conversely, an RR of less than 1.0 indicates that the variable has a protective effect. (The OR is used to estimate the RR under certain conditions.)
- The article reported the number of cases and non-cases (i.e., controls in case-control studies and person-time exposed during the followup in a cohort study) and estimates of the RR or OR for each exposure level.

Two readers, who received no information on the names and affiliations of the authors of each study or the alcohol-related results, independently determined the eligibility of each article for inclusion in the meta-analysis. When the results of a study were published in more than one article, only the most recent and complete article was included in the analysis.

To estimate the effect of alcohol consumption on the risk for each type of cancer studied, based on the pooled data from all studies included in the meta-analysis, the investigators used meta-regression models—statistical models developed specifically for such analyses (Corrao et al. 1999, 2000). For a more detailed description of these statistical analyses, see the textbox, p. 265, and the articles by Corrao and colleagues (1999, 2000).

**Statistical Methods Used in the Meta-Analysis**

To determine the effects of alcohol on the risk for various types of cancer, the researchers used three statistical methods. They first pooled the original published data for each type of cancer. Subsequently, they determined the relationship between alcohol consumption and the risk for a given type of cancer by fitting to the pooled data several statistical models called fractional models (Royston et al. 1999). Such models can identify trends (e.g., J- or U-shaped curves) as well as other relationships between alcohol exposure levels and relative risks. The investigators then chose the best-fitting model to summarize the relation of interest (Corrao et al. 1999). Next, they assessed whether gender modified the effect of alcohol on the risk for each neoplasm. They also looked at the effect of adjusting the reported estimates for smoking when examining tobacco-related types of cancer. Finally, the researchers evaluated the variability (i.e., heterogeneity) among the studies’ results according to methods proposed by Greenland and Longnecker (1992).

## Results of the Meta-Analysis

In all, 229 studies (183 case-control studies and 46 cohort studies) met the eligibility criteria and were included in the meta-analysis. These studies, which reported a total of 115,199 cases, investigated alcohol’s effects on the risk for developing cancer at a total of 19 sites in the body or at all sites combined (see the table and figure for a summary of the studies and their findings for each of those sites). For five of the cancer sites (i.e., small intestine, gall bladder, skin [melanoma], cervix, and kidney), alcohol’s effects were analyzed in only one or two studies; in these cases, the researchers could not calculate the pooled RR or 95-percent confidence interval (95% CI).^2^ For all other cancer sites, both the pooled RR and the 95% CI were determined for three levels of alcohol consumption (i.e., 25, 50, and 100 grams of alcohol per day, corresponding to approximately 2, 4, and 8 standard drinks^3^ per day, respectively).

For most of the tumor types included in the studies, the analysis found a dose-dependent increase in risk from alcohol consumption—that is, greater alcohol consumption was associated with a greater increase in risk. Alcohol most strongly increased the risks for cancers of the oral cavity and pharynx (RR=5.7 for the highest alcohol consumption level), esophagus (RR=4.2) and larynx (RR=3.2). Appreciably smaller, although still statistically significant at the 5-percent level,^4^ increases in risk existed for cancers of the stomach, colon, rectum, liver, female breast, and ovaries. The smallest increases in risk were observed for cancers of the lung (RR=1.1 at the highest consumption level) and prostate (RR=1.2). For all these types of cancer, significant increases in risk existed even at the lowest consumption level studied here (i.e., 25 grams of alcohol, or two standard drinks per day). In contrast, no significant relationship existed between alcohol consumption and the risk for pancreas, endometrial, and bladder cancers. With the exceptions of cancers of the ovary, prostate, and bladder, significant heterogeneity across studies existed for each type of cancer; this means that results vary greatly among the various studies analyzed, so an overall summary of average effect across studies must be taken with caution.

The researchers also investigated whether gender modified the effect of alcohol intake on the risk for each type of cancer. Statistically significant gender differences existed only for esophageal and liver cancer—where the alcohol-related risk was higher in women than in men—but not for other types of cancer.

The results of eight appropriate studies were pooled to determine the relationship between alcohol consumption and the risk of cancer at all sites combined. This analysis found that alcohol consumption of at least 50 grams (i.e., 4 standard drinks) per day significantly increased the risk of developing any type of cancer.

## Discussion of the Study Findings

### Limitations and Strengths of the Meta-Analysis

As with other meta-analyses of published studies, the analysis presented here has various limitations and strengths. One limitation is that for most types of cancer included, the estimates of alcohol’s effects tended to vary widely among the individual studies, making interpretation of the pooled data more difficult. Part of this variability may result from differences in the characteristics of the subjects included in the studies. For example, the gender of the study participants may play a role because potential differences in alcohol breakdown (i.e., metabolism) exist between men and women and may systematically influence the overall pooled estimates (Corrao et al. 1999, 2000).

To control for this possibility, the investigators included separate analyses for men and women in their statistical models, where feasible. However, gender explained a significant portion of the observed variability in study results only for esophageal and liver cancer, but not for other types of cancers. Another limitation of this and other meta-analyses is that alcohol consumption levels may have been systematically underreported in several studies, leading to biased RR estimates.

The statistical power of this meta-analysis also is somewhat limited because, although it included more than 115,000 cases, the numbers of cases for certain types of cancers (e.g., cervix and melanoma) were relatively small. Similarly, for certain cancers, there were only a few cases with certain levels of alcohol consumption (e.g., high levels of alcohol consumption in female breast cancer patients). Furthermore, biases resulting from confounding factors may have affected the pooled estimates. A confounding factor is a variable that is related to both the exposure variable (e.g., alcohol consumption) and the risk of disease (e.g., cancer). For example, alcohol intake may appear to be positively associated with lung cancer but the actual association may be confounded by cigarette smoking, which is related with both alcohol intake (because people who smoke also tend to drink) and the risk of lung cancer.

**Table:** Summary of the Studies Included in the Meta-Analysis

Cancer site	No. of Studies	No. of Cases	Study design		Pooled RR [and 95% CI] associated with alcohol intake of			Gender effect (*p*)	Heterogeneity test (*p*)
	
			Cohort	Case-control	25 g/day [range]	50 g/day [range]	100 g/day [range]		
Oral cavity	26	7,954	1	25	1.73 [1.67–1.78]	2.77 [2.67–2.95]	5.75 [5.22–6.34]	n.s.	< 0.01
Esophagus^a^	28	7,239	1	27	1.51 [1.48–1.55]	2.21 [2.11–2.31]	4.23 [3.91–4.59]	< 0.05	< 0.01
Males	18	3,310	1	17	1.43 [1.38–1.48]	1.98 [1.87–2.11]	3.49 [3.14–3.89]	-	< 0.01
Females	5	304	0	5	1.52 [1.42–1.63]	2.24 [1.95–2.58]	4.45 [3.37–5.87]	-	
Stomach	16	4,518	2	14	1.07 [1.04–1.10]	1.15 [1.09–1.22]	1.32 [1.18–1.49]	n.s.	< 0.01
Small intestine	2	415	0	2	- -	- -	- -	-	-
Colon	17	5,948	4	13	1.14 [1.07–1.21]	1.21 [1.11–1.32]	1.32 [1.16–1.49]	n.s.	< 0.01
Rectum	16	3,872	3	13	1.11 [1.03–1.20]	1.17 [1.06–1.30]	1.32 [1.16–1.51]	n.s.	< 0.01
Liver^a^	19	1,961	3	16	1.20 [1.13–1.27]	1.41 [1.26–1.56]	1.83 [1.53–2.19]	< 0.05	< 0.01
Males	10	949	2	8	1.28 [1.13–1.45]	1.51 [1.27–2.10]	1.62 [1.18–2.24]	-	< 0.01
Females	3	231	1	2	1.97 [1.30–3.00]	3.57 [1.56–8.21]	9.15 [1.73–48.41]	-	
Gallbladder	2	81	1	1	- -	- -	- -	-	-
Pancreas	17	2,524	4	13	0.98 [0.90–1.05]	1.05 [0.93–1.18]	1.18 [0.94–1.49]	n.s.	< 0.01
Larynx	20	3,759	0	20	1.35 [1.31–1.40]	1.83 [1.72–1.95]	3.24 [2.89–3.65]	n.s.	< 0.01
Lung	6	2,314	3	3	1.02 [1.00–1.04]	1.04 [1.00–1.08]	1.08 [1.00–1.18]	n.s.	< 0.01
Melanoma	2	708	0	2	- -	- -	- -	-	-
Female Breast	49	44,033	12	37	1.31 [1.27–1.36]	1.67 [1.56–1.78]	2.71 [2.33–3.08]	-	< 0.01
Cervix	1	242	-	1	- -	- -	- -	-	-
Endometrium	6	2,473	2	4	1.05 [0.88–1.24]	1.09 [0.78–1.54]	1.20 [0.60–2.37]	-	<0.01
Ovary	5	1,651	-	5	1.11 [1.00–1.24]	1.23 [1.01–1.54]	1.53 [1.03–2.32]	-	n.s.
Prostate	11	4,094	4	7	1.05 [1.00–1.08]	1.09 [1.02–1.17]	1.19 [1.03–1.37]	-	n.s.
Bladder	11	5,997	2	9	1.04 [0.99–1.09]	1.08 [0.98–1.89]	1.17 [0.97–1.41]	n.s.	n.s.
Kidney	2	921	0	2	- -	- -	- -	-	-
All sites combined	8	14,495	6	2	1.01 [0.90–1.05]	1.22 [1.11–1.27]	1.91 [1.77–2.06]	n.s.	< 0.01
Total*	229	115,199	46	183	-	-	-	-	-

NOTE: Table gives the pooled relative risks (RR) and corresponding 95-percent confidence intervals (95% CI) for three alcohol consumption levels. Those levels specified corresponded to approximately two, four, and eight standard drinks per day, respectively. The effects of gender and differences (i.e., heterogeneity) in the study results for various cancer sites also are presented. The RR indicates the strength of the relationship between alcohol consumption and a given type of cancer. A RR greater than 1.0 means that alcohol consumption at the level indicated increased the risk for that type of cancer. The greater the value over 1.0, the greater the risk. The 95-percent confidence intervals indicate the range of RR that is 95 percent likely to show a true RR. A statistically significant heterogeneity level indicates that results varied greatly among the various studies analyzed and that, therefore, an overall summary of average effect across studies must be interpreted with caution.

* The number of individual studies does not add up to the total shown because several studies examined more than one type of cancer.

a For this type of cancer, the RRs associated with alcohol consumption for males and females are listed separately because a statistically significant difference existed between the two sexes.

n.s. = not significant

One of the strengths of this meta-analysis is that the investigators performed a separate analysis of studies that also reported estimates adjusted for tobacco use, which contributes to various forms of cancer, prominently lung cancer. Such analyses were conducted for most cancers of the upper airways and digestive tract, as well as for lung and bladder cancer. These analyses found that tobacco use had a substantial modifying effect not only on the alcohol-related risks for lung and bladder cancer but also on the risk for laryngeal cancer. For example, when the investigators considered only studies reporting RRs not adjusted for tobacco use, the pooled RR for lung cancer at the highest level of alcohol consumption was 6.30. When they excluded such studies from the analysis and considered only studies reporting estimates adjusted for tobacco use, however, the pooled RR declined to 1.07. This finding indicates that alcohol itself only weakly increases the risk for lung cancer and that lung cancer risk primarily results from tobacco use, which is common in heavy drinkers. For laryngeal cancer, tobacco use also substantially influences the risk, though a strong association with alcohol consumption, indicated by a RR of 3.24, remained even when considering only studies presenting adjusted estimates.

The association between various levels of alcohol consumption and an increased risk of liver cancer remains difficult to interpret even with the pooled data used in this meta-analysis. This difficulty results from the fact that, as discussed earlier, the association between alcohol consumption and liver cancer is only indirect. Furthermore, patients with liver cancer resulting from cirrhosis typically have reduced their alcohol consumption by the time they develop liver cancer (Aricò et al. 1994). This change in consumption levels may lead to an underestimate of the real association.

### Mechanisms of Alcohol-Related Carcinogenesis

Researchers have known about the relationship between heavy alcohol consumption and the risk for esophageal and other upper digestive and respiratory tract cancers since the beginning of the last century. Furthermore, substantial epidemiological evidence (as reviewed in this article) accrued over the past 50 years has shown that alcohol contributes to the development of these cancers. Nevertheless, the mechanisms underlying alcohol-related cancer development remain largely unclear.

To date, no experimental evidence indicates that alcohol by itself can cause cancer—that is, that alcohol can act as a complete carcinogen. Over the past few decades, however, several animal studies have indicated that alcohol can have a cocarcinogenic, or cancer-promoting, effect. This means that when alcohol is administered together with other known cancer-inducing agents (i.e., carcinogens), it promotes or accelerates cancer development. This effect was noted for several digestive tract cancers, specifically cancers of the esophagus and the nonglandular forestomach^5^ (Doll et al. 1999).

For other cancers of the digestive tract (e.g., stomach, pancreas, colon, and rectum), however, the results are less clear and generally are variable across studies, possibly because of differences in study design. Researchers also have no clear understanding of the potential mechanisms through which alcohol might act as a cocarcinogen at these sites (IARC 1988; Doll et al. 1999). Moreover, the RR estimates based on the pooled data in this meta-analysis ranged from 1.1 to 1.3 for the highest level of alcohol intake. These values indicate only a weak association of alcohol with these types of cancer, which may possibly result from residual bias in the analysis or from confounding factors, such as diet. Therefore, one cannot draw any conclusions regarding a potential causal role of alcohol in the development of these cancers.

For female breast cancer, the meta-analysis described here confirms the existence of a strong dose-risk relationship between alcohol consumption level and breast cancer risk. The exact role of alcohol in the development of breast cancer remains unclear. It is possible, however, that for breast cancer and other types of cancer related to disturbances in female hormone levels, alcohol may act by altering the metabolism and blood levels of female hormones, such as estrogen (Longnecker 1994). Moreover, a recent study suggests that the association may be limited to women with a family history of breast cancer (Vachon et al. 2001).

### Effects of Combined Alcohol and Tobacco Use

Alcohol and tobacco enhance each other’s effects (i.e., act synergistically) on the risk for cancers of the upper digestive and respiratory tract.^6^ Studies conducted in North America, France, and Italy have shown extremely high RRs for certain cancers among people who both drink and smoke heavily (Doll et al. 1999). For example, in a series of case-control studies conducted in Italy, the RRs for the highest exposure levels to both risk factors were 80 for cancers of the oral cavity and pharynx, 12 for laryngeal cancer, and 18 for esophageal cancer (Franceschi et al. 1990). From a public health view, this synergism implies that over 75 percent of cancers of the upper digestive and respiratory tract in developed countries are attributable to alcohol and tobacco. Accordingly, the cessation or moderation of tobacco and/or alcohol use could avoid the majority of these cancer cases.

**Figure:**
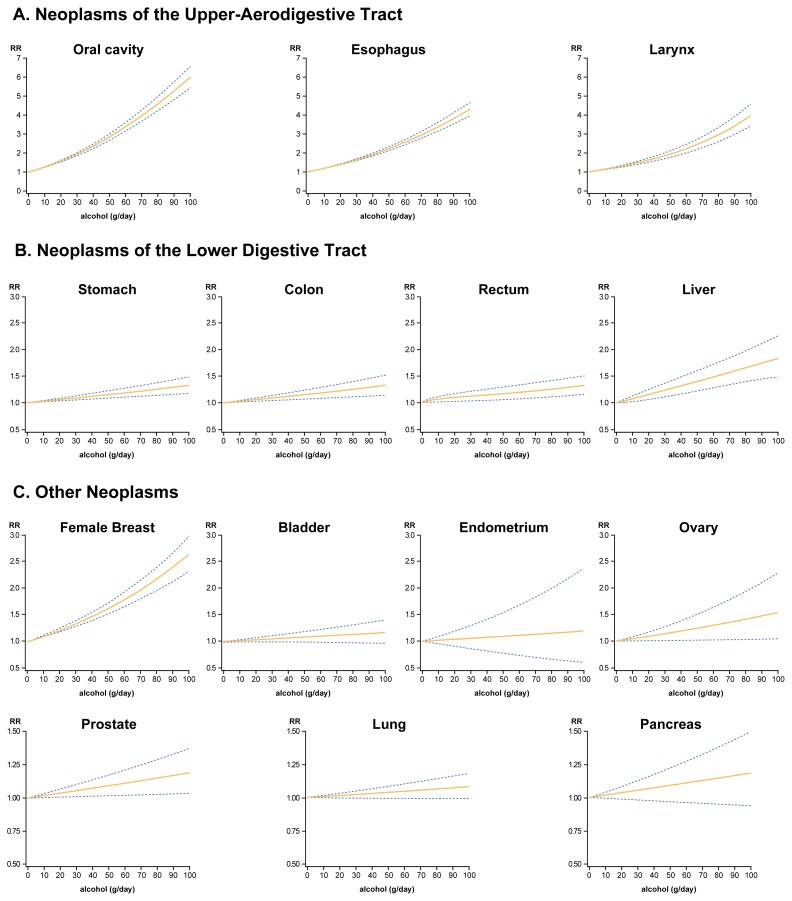
Relationship between increasing amounts of alcohol and risk (i.e., relative risk or RR) for 14 types of cancer. The RR describes the strength of the relationship between a variable (e.g., alcohol consumption) and a disease (e.g., cancer). The RR for the disease in people without the variable (e.g., abstainers) is defined as 1.0. A RR among the people with the variable (e.g., drinkers) of greater than 1.0 indicates that the variable increases the risk for the disease. The greater the value, the greater the risk. The curves shown here were obtained by fitting certain statistical models to the data from several studies (i.e., a meta-analysis). Blue dotted lines indicate 95-percent confidence intervals; that is, the range of RR that is 95 percent likely to show a true RR.

The synergism between alcohol and tobacco also implies that cancers of the upper digestive and respiratory tract in Europe and North America are extremely rare among people who have never smoked, and only limited data are available on the effects of alcohol in those “never-smokers.” Those limited data indicate that for oral and pharyngeal cancers (Fioretti et al. 1999; Talamini et al. 1998) as well as for esophageal cancer (Doll et al. 1999), high alcohol consumption in people who have never smoked is directly associated with an increased risk for these types of cancer. (For laryngeal cancer, the evidence in this respect is inconsistent.) The substances contained in tobacco products are not the only carcinogenic agents to which the upper digestive tract is exposed, however, and alcohol may facilitate or promote the effects of other carcinogens present in the diet or other environmental exposures.

### Risks Associated with Different Types of Alcoholic Beverages

Researchers have made several attempts to determine whether different types of alcoholic beverages have different effects on cancer risk. Some studies found no apparent differences in the cancer risks associated with various beverages, whereas others have reported greater risks with spirits than with wine or beer (Doll et al. 1999). Researchers conducting a study in Normandy, France (Tuyns et al. 1979), reported an increased risk of esophageal cancer associated with apple-based drinks (e.g., apple brandy and hard cider). Two studies from Italy (Barra et al. 1990; Bosetti et al. 2000) found that people who consumed only wine had greater risks of oral, pharyngeal, and esophageal cancer compared with people who consumed wine as well as distilled spirits or beer after adjusting for the overall amount of alcohol consumed. Conversely, a Danish study found no excess risk for cancer of the upper digestive tract associated with wine consumption (Gronbaek et al. 1998).

Taken together, these findings appear to indicate that the most frequently consumed beverage in each region or country tends to be the beverage associated with the highest estimated RR. However, this topic requires further study. Some researchers have suggested that drinks containing higher alcohol concentrations are more deleterious per gram of alcohol than drinks with lower alcohol concentrations. The evidence for this hypothesis, however, is weak and inconclusive (Doll et al. 1999).

## Conclusions

This meta-analysis includes most published information on alcohol and cancer and, the limitations discussed above notwithstanding, consequently provides the most accurate estimates of the RRs for common cancers considered to be alcohol-related. Some of the findings are novel and of specific relevance. For example, the analysis was unable to identify a threshold level of alcohol consumption below which no increased risk for cancer is evident. Furthermore, this meta-analysis found that the association of alcohol with the risk for oral and pharyngeal cancer appears to be stronger than the association with esophageal or laryngeal cancer across increasing levels of alcohol intake.

In terms of risk assessment, this meta-analysis confirms that high levels of alcohol consumption (i.e., more than four drinks per day) result in a substantial risk of cancer development at several sites. Lower levels of consumption result in a moderately increased risk for various cancers. At the same time, other studies have shown that moderate alcohol consumption can have protective effects against certain types of heart disease. Accordingly, one must determine whether moderate alcohol consumption results in an overall favorable or unfavorable risk-benefit balance for the individual drinker or an entire population. This balance depends on the age, gender, and baseline disease rates among the members of a given population. In addition, genetic factors also may influence a person’s risk-benefit balance as suggested by the previously mentioned findings that the association between alcohol consumption and female breast cancer may be limited to women with a family history of breast cancer (i.e., with predisposing genetic factors) (Vachon et al. 2001). Consequently, any definite risk-benefit assessment for moderate alcohol drinking requires much more far-reaching analyses that are beyond the scope of this article but that in the future may provide important information from a public health perspective.

## Footnotes

1 The definitions of light, moderate, and heavy alcohol consumption vary across studies. In the United States, the U.S. Department of Health and Human Services and the U.S. Department of Agriculture have defined moderate drinking as no more than one drink per day for women and no more than two drinks per day for men.

2 Statistical analyses generally do not lead to a calculated, accurate result but instead provide an estimate of the result. The 95% CI is the range of the value under investigation that with a 95-percent likelihood contains the true value.

3 In the United States, a standard drink frequently is defined as 0.5 ounces (oz) or 14 grams of pure alcohol. This alcohol amount is found in 12 fluid oz of beer, 5 fluid oz of wine, and 1.5 fluid oz of 80-proof distilled spirits.

4 Statistically significant means that the observed result would occur by chance under hypothesized conditions less than a specified proportion of the time (usually 5 percent).

5 These studies were conducted in hamsters. The stomach of a hamster is divided into a portion that does not contain glands which secrete digestive enzymes (i.e., nonglandular forestomach) and a portion closer to the intestine that does contain such glands.

6 Similarly, synergistic effects have been reported between high alcohol consumption and dietary factors, such as low consumption of fruits and vegetables.
